# ctDNA-derived copy-number signatures associated with immune checkpoint inhibitor resistance beyond PD-L1 and TMB in advanced NSCLC

**DOI:** 10.3389/fonc.2026.1876274

**Published:** 2026-07-31

**Authors:** Pedro Gonzalez Santa-Catalina, Luis Posado-Domínguez, Álvaro López-Gutiérrez, Edel del Barco Morillo, Juan Carlos Redondo-González, Noelia Egido-Iglesias, Laura Corvo-Félix, Isabel Muela Bravo, Aline Rodrigues-Françoso, Lorena Bellido-Hernández, Emilio Fonseca-Sánchez, Juan Jesús Cruz-Hernández, Juan Manuel Corchado, Juan Luis García Hernández, Alejandro Olivares-Hernández

**Affiliations:** 1 Medical Oncology Department, Salamanca University Hospital, Salamanca, Spain; 2 Biomedical Research Institute of Salamanca (IBSAL), Salamanca, Spain; 3 Cancer Research Centre (CIC), Salamanca, Spain; 4 Faculty of Medicine, University of Salamanca, Salamanca, Spain; 5 Research Group on Bioinformatics, Intelligent Computer Systems, and Educational Technology (BISITE) Research Group, University of Salamanca, Salamanca, Spain; 6 Air Institute, IoT Digital Innovation Hub, Salamanca, Spain; 7 Department of Electronics, Information and Communication, Faculty of Engineering, Osaka Institute of Technology, Osaka, Japan; 8 Computing and Information Technology Center, Malaysia Kelantan University, Bachok, Kelantan, Malaysia

**Keywords:** copy-number signatures, ctDNA (circulating tumour DNA), immunotherapy, mutational signature, non-small cell lung cancer, PD-L1, tumour mutational burden (TMB), whole-exome sequencing (WES)

## Abstract

**Background:**

Immune checkpoint inhibitors (ICIs) have improved outcomes in advanced non-small cell lung cancer (NSCLC), but clinical benefit remains heterogeneous and is not fully explained by programmed death-ligand 1 (PD-L1) expression or tumour mutational burden (TMB). We explored whether whole-exome sequencing (WES) of circulating tumour DNA (ctDNA) could identify mutational and copy-number (CN) signatures associated with outcome in advanced NSCLC treated with first-line ICI.

**Methods:**

Baseline plasma ctDNA from 37 patients with advanced NSCLC treated with first-line ICI or chemo-ICI was analysed by WES. Single-base substitution (SBS), indel (InD) and CN signatures were inferred using COSMIC-based signature frameworks. SBS signatures reflect point mutation patterns, including clock-like processes that accumulate with age; indel signatures reflect small insertions and deletions; and CN signatures summarise structural changes such as chromosomal gains and loss of heterozygosity. Signatures were integrated with PD-L1 status, TMB, progression-free survival (PFS) and overall survival (OS). Patients were classified into four clinicogenomic groups according to PD-L1 expression and PFS.

**Results:**

Median PFS and OS were 9 and 20 months, respectively. Single-nucleotide-variant-derived TMB (TMB-SNV) was similar in patients with adverse and favourable outcomes, and patients with TMB-SNV >10 mutations/Mb were evenly distributed between both groups. The SBS profile was mainly represented by the clock-like signatures SBS1, SBS5 and by the tobacco-like signature SBS92, whereas indel signatures were mostly represented by InD4a and InD10. CN signatures showed the most apparent separation between outcome groups. CN9 exposure, a marker of structurally unstable genomes, was higher in adverse-outcome patients and most prominent in Group 1 (PD-L1 ≥50% and adverse outcome), whilst CN21 exposure was highest in Group 4 (PD-L1 ≥50% and favourable outcome). No signature retained statistical significance after correction for multiple testing.

**Conclusions:**

In this exploratory cohort, ctDNA-derived CN signatures may capture structural genomic phenotypes associated with ICI outcome beyond PD-L1 and TMB. CN9 emerged as a candidate resistance-associated signature, whereas CN21 was associated with more favourable disease control. These findings are hypothesis-generating and require validation in larger prospective cohorts.

## 1 Introduction

Immune checkpoint inhibitors (ICIs), administered either as monotherapy or in combination with chemotherapy, constitute a cornerstone of first-line treatment for advanced non-small cell lung cancer (NSCLC) without actionable driver molecular alterations (1, 2). Despite this major therapeutic advance, clinical benefit remains heterogeneous. A subset of patients achieves deep responses and disease control lasting several years, whereas others show primary resistance or early progression in the presence of apparently favourable biomarker profiles (3). In current practice, programmed death-ligand 1 (PD-L1) expression remains the most widely used biomarker for guiding ICI-based treatment, whilst tumour mutational burden (TMB) has been investigated as a complementary indicator of immunogenicity (4, 5). Neither parameter, however, fully captures the complexity of tumour and immune interactions, genomic instability, or the evolutionary dynamics that underlie immune escape (3, 5).

This limitation is especially significant in advanced NSCLC, where tumour biology reflects multiple endogenous and exogenous mutational processes; for instance, tobacco exposure, cellular ageing, defects in deoxyribonucleic acid (DNA) repair, apolipoprotein B mRNA editing catalytic polypeptide-like (APOBEC) enzyme activity, chromosomal instability and clonal selection have been found to each contribute to tumour development andtherapeutic resistance (6, 7). In this setting, mutational signature analysis offer a potential framework for the study of such processes by identifying recurrent patterns of genomic damage (7). Single-base substitution signatures (SBS) reflect characteristic nucleotide changes; insertion and deletion signatures (indel, InD) capture small gains or losses of bases; copy-number (CN) signatures summarise broader structural alterations, including chromosomal gains or losses, allelic imbalance, and loss of heterozygosity (LOH) (7–9). Considered jointly, these signatures may provide a more integrated view of tumour evolution than approaches based solely on individual genes or on TMB (8, 9).

In NSCLC, several mutational signatures have been associated with clinically relevant processes, including tobacco-related DNA damage, ageing or clock-like signatures, APOBEC-mediated mutagenesis, mismatch repair deficiency (MMRd), and homologous recombination deficiency (HRD) (6, 7, 10), which may modulate tumour immunogenicity, sensitivity to platinum-based chemotherapy, response to ICIs, and patterns of acquired resistance (10, 11). Nevertheless, most available evidence comes from tumour tissue sequencing, whereas the clinical utility of mutational signatures obtained from circulating tumour DNA (ctDNA) remains less well defined. This gap is relevant because liquid biopsy constitutes a minimally invasive approach for the assessment of tumour heterogeneity in advanced disease, particularly in scenarios with limited or unavailable tissue (12).

Whole exome sequencing (WES) of ctDNA extends this framework by allowing simultaneous evaluation of single nucleotide variants (SNVs), InDs, copy-number variations (CNVs), and global mutational patterns. When integrated with advanced bioinformatic tools, this approach may reveal genomic phenotypes associated with sensitivity or resistance to ICIs beyond PD-L1 and TMB (11, 13). Among the signature classes, CN signatures may represent a structural dimension of genomic instability that point mutation burden alone reflects poorly (8, 9, 14, 15). Distinct CN signatures are thought to arise from different mechanisms of chromosomal instability, and they need not carry the same prognostic meaning, since some patterns reflect marked structural disruption whilst others accompany more stable disease (8, 9).

In this exploratory study, we analysed baseline ctDNA samples from patients with advanced NSCLC treated with first-line immunotherapy (IO) or chemo-ICI. We combined SBS, InD and CN signatures with PD-L1, blood-based TMB (bTMB), and clinical outcomes, including progression-free survival (PFS) and overall survival (OS). The aim was to determine whether ctDNA-derived mutational signatures could identify genomic phenotypes linked to response or resistance to ICI and provide a rationale for the development of composite biomarker models in advanced NSCLC.

## 2 Materials and methods

### 2.1 Study design and population

This was an observational, prospective, exploratory, single-centre study including patients with advanced NSCLC treated at the Medical Oncology Department of the University Hospital of Salamanca, Spain. The study was conducted in collaboration with the BISITE Research Group of the University of Salamanca and the Cancer Research Center (CIC).

Patients were eligible if they were aged ≥18 years, had histologically confirmed advanced NSCLC, no prior exposure to ICI, and absence of classical actionable driver alterations, including activating *EGFR* mutations or *ALK*/*ROS1* rearrangements. All patients received first-line ICI-based treatment either as monotherapy or in combination with chemotherapy, in accordance with standard practice and current treatment guidelines (16). Availability of a baseline peripheral blood sample for liquid biopsy before treatment initiation was mandatory. Exclusion criteria comprised previous ICI exposure, history of another malignancy requiring systemic treatment, presence of exclusionary actionable driver alterations, and peripheral blood collection after the start of ICI. Individual clinical, demographic, and signature-level data for the 37 patients are provided in **Supplementary Table 1**.

### 2.2 Clinical variables and outcome definitions

Clinical, demographic, histological and molecular variables were retrieved from electronic medical records. The dataset comprised age, sex, smoking status, tumour histology, disease stage, PD-L1 expression, molecular alterations detected in routine practice, presence of brain metastases, first-line treatment received, immune-related toxicity, toxicity grade according to CTCAE v5.0 (applied to standardise toxicity grading and ensure comparability with contemporary clinical trials and real-world studies), radiological response according to RECIST v1.1, objective response rate (ORR), PFS, and OS. ORR was defined as the sum of complete and partial responses according to RECIST v1.1; PFS, as the interval from initiation of first-line systemic treatment to radiological progression, death or last follow-up; and OS, as the interval from treatment initiation to death from any cause or last follow-up.

For the clinicogenomic analysis, patients were stratified according to two predefined variables: PD-L1 expression and clinical outcome defined by PFS. PD-L1 expression was categorised as high when ≥50% and low when<50%. Clinical outcome was classified as favourable or adverse using a PFS cut-off of 9 months, selected for its clinical relevance in first-line ICI for advanced NSCLC and according to the available sample size. Four clinicogenomic groups were defined: Group 1 (adverse outcome and PD-L1 ≥50%), Group 2 (adverse outcome and PD-L1<50%), Group 3 (favourable outcome and PD-L1<50%), and Group 4 (favourable outcome and PD-L1 ≥50%). For signature-level analyses, outcome was additionally treated as a binary variable distinguishing adverse from favourable cases, which underlay all between-group comparisons of signature exposure. This classification was applied to investigate whether ctDNA-derived genomic signatures could identify resistance or sensitivity phenotypes not explained by PD-L1 alone.

### 2.3 ctDNA collection and processing

Peripheral blood was collected before initiation of systemic treatment. In most patients, blood sampling was performed on the same day as treatment initiation or within a pre-treatment window of up to 10 days. Two 10 mL EDTA tubes of peripheral blood were obtained. Plasma was isolated by initial centrifugation of whole blood for 10 minutes at 1,900 × g, followed by a second centrifugation for 10 minutes at 18,000 × g in the presence of EDTA and proteinase K. ctDNA was subsequently extracted using the QIAamp Circulating Nucleic Acid Kit. Samples were stored at −80 °C until further processing. In the original protocol, approximately 20–80 ng of ctDNA were obtained from 4–5 mL of plasma.

### 2.4 WES and bioinformatic processing

Genomic profiling was performed using WES of baseline plasma-derived ctDNA. Exome library preparation was performed using the SureSelect Human All Exon V6 capture kit. Sequencing was carried out on a NovaSeq6000 platform using paired-end 150 × 2 bp reads. The average sequencing output was approximately 18 Gb per sample. Library preparation and quantification were performed by Macrogen Inc.

Sequencing data were analysed using the AIRGenomics platform, which integrates quality control, read alignment, variant calling, annotation, and clinical prioritisation within a single workflow (17). Raw reads were initially evaluated for base quality, sequencing depth, and potential sample contamination. Reads passing quality thresholds were aligned to the human reference genome (hg19) through algorithms suited for somatic variant detection at low allele frequencies. Variant calling included SNVs, InD, and structural variants, followed by annotation against reference databases such as ClinVar, gnomAD, and dbSNP. SNVs and indels were processed separately for manual review. Only SNVs/InD labelled as PASS were retained, whilst for InD both ‘PASS’ and ‘LowQual’ variants were considered; variants marked as ‘REJECTED’ were excluded. Additional manual filtering criteria were applied to both variant types.

### 2.5 Mutational signature analysis

CN profiles were derived from the same WES data. Genome-wide log2 coverage ratios were segmented, and segment-level calls were classified using log2-ratio thresholds corresponding to CN 0 (≤ −1.1), CN 1 (≤ −0.25), CN 2 (≥ 0.2), and CN 3 (≥ 0.7). Within this scheme, segments below the diploid reference defined CN losses, encompassing both single-copy loss and deeper deletions, whereas segments above the diploid reference defined CN gains; this gain–loss distinction was preserved as input to CN signature inference. Only variants with consistent support across methods and meeting quality criteria were included in the final analysis.Mutational signature analysis.

Somatic variants were subjected to stringent filtering prior to downstream analysis. Retention required minimum sequencing depth ≥100×, variant allele frequency (VAF) ≥0.5–1% depending on sample quality, base quality ≥Q30, and absence of significant strand bias or sequencing artefacts. To minimise confounding from non-tumour-derived mutations, variants located in canonical clonal hematopoiesis (CH)-associated genes (*DNMT3A*, *TET2*, *ASXL1*, *JAK2*, *PPM1D*, *SF3B1*, and *SRSF2*) were excluded. This step is relevant in ctDNA analyses, especially in older patient cohorts, where CH-derived mutations may contribute to background noise and inflate age-related mutational processes.

Genomic signature analysis was performed using somatic alterations identified from ctDNA WES, evaluating three classes of signatures: SBS, InD, and CN. SBS signatures were defined according to the type of base substitution and its trinucleotide sequence context, whereas InD signatures were classified according to event length, repetitive sequence context, and local sequence characteristics. SBS and InD signature analysis was performed using Signal through the FitMS framework, which estimates the contribution of previously described mutational signatures to individual samples (18, 19).

CN signatures required a separate workflow. First, CN profiles were generated from WES data with CNVkit. These profiles were then analysed with SigProfilerAssignment, through COSMIC Mutational Signatures web platform (https://cancer.sanger.ac.uk/signatures/), which describes each CN profile using a 48-feature scheme based on segment length, total CN state, LOH, and allelic composition (7, 8). Established COSMIC reference signatures were fitted to each sample rather than extracted *de novo*. This approach was adopted because the cohort was small (n = 37), and the fitting of known signatures yields more stable and reproducible results than the extraction of new ones. Fitting relied on a sparse optimisation approach that combines forwards stagewise regression with non-negative least squares, which estimates the activity of each reference signature within a sample. Cosine similarity was additionally calculated as a measure of fit between the observed mutational profile and the pattern reconstructed from the assigned signatures. This metric was used to assess the stability of the signature model and to investigate whether dominance of a restricted mutational process was associated with adverse clinical outcome.

For every sample, an exposure value was obtained for each SBS, InD, and CN signature. Each exposure reflects the relative weight of a mutational process within that sample, not a mutation count or a mutations-per-megabase value. Concordance between the signatures derived from point mutations (SBS/InD) and those derived from CN changes was then assessed to confirm that both reflected consistent underlying processes. The resulting signature exposure matrices were integrated with clinical and molecular variables, including PD-L1 expression, PFS, and OS, for downstream statistical analyses aimed at identifying molecular phenotypes associated with response to ICI therapy.

### 2.6 bTMB and tumour fraction

bTMB was calculated as the number of somatic mutations per megabase analysed. For descriptive analyses, SNV-derived TMB (TMB-SNV) and indel-derived TMB (TMB-indel) were reported separately. bTMB was subsequently integrated with PD-L1 status, PFS, OS and ctDNA-derived genomic signatures to explore its value as a standalone biomarker and in combination with additional molecular features.

The tumour fraction was computationally estimated based on the allele frequencies of the somatic variants identified in each sample. To minimise the confounding impact of sequencing artefacts, subclonal mutations, or localised copy number alterations, the median VAF across all filtered variants was calculated for each specimen. Assuming a generalised heterozygous state in diploid genomic regions, the total tumour fraction was formally derived using the following equation:


Tumour Fraction=Median VAF x 2

The resulting values were expressed as both decimal fractions and percentages to define sample purity and facilitate subsequent statistical analysis.

### 2.7 Hierarchical clustering and heatmap visualisation

An exploratory unsupervised analysis was performed using heatmaps and hierarchical clustering to evaluate the distribution of SBS, indel and CN signatures across patients. Heatmaps were generated in Python (seaborn and matplotlib packages), based on the relative contribution of each signature per sample, with variable scaling applied to facilitate visual comparison across signatures. An initial global heatmap encompassed the broader set of available signatures. A second reduced heatmap focused on signatures with higher variability or greater clinical interest, comprising SBS1, SBS5, SBS92, InD4a, CN1, CN9, CN21 and CN25, chosen on the basis of their relative contribution, distribution across samples, and exploratory association with clinicogenomic groups.

The analysis script performed an exploratory workflow on the ctDNA cohort and generated two main outputs: a hierarchically clustered heatmap of selected signatures and a standardised median-Δ plot. The script first loaded the dataset, cleaned column names, converted numeric fields, and identified the outcome variable as ‘Response’, which contained the two groups ‘favourable’ and ‘adverse’ (**Supplementary Table 1**). The available signatures were separated into SBS, InD, and CN classes, and only signatures with more than 5 non-missing values were retained for downstream analysis.

#### 2.7.1 Signature selection for the reduced heatmap

The reduced heatmap was built from the intersection of two exploratory filters: the 10 signatures with the highest variance across the cohort and the 10 signatures with the smallest Mann–Whitney U test p-values comparing ‘favourable’ versus ‘adverse’. The final selected set used for the reduced heatmap was: SBS1, SBS92, SBS5, InD4a, CN1, CN9, CN21, and CN25. This selection was therefore based on variability and exploratory group separation, rather than a formal supervised feature-selection procedure. CN1 was retained because it showed evaluable contribution across the cohort and formed part of the biologically informative CN pattern represented in the reduced panel.

To support biological interpretation, each signature retained for the reduced analysis was assigned an annotation based on established aetiologies. SBS1 and SBS5 are clock-like signatures linked to endogenous ageing (20). SBS92 is tobacco-associated; because the canonical tobacco signature in lung cancer is SBS4, SBS92 is reported here as tobacco-like in this dataset (21). InD4a is an InD signature related to topoisomerase I-associated processes (22). CN1 reflects a near-diploid genome with few CN changes, whereas CN9 reflects loss of heterozygosity and chromosomal instability (8). CN21 is a CN signature whose biological cause is not yet established. CN25, a minor signature detected in a small subset of samples, is shown in the integrated panel without individual biological annotation. CN signatures such as CN9 and CN21 should be understood as computational patterns reflecting structural genomic processes, not as individual genes or specific chromosomal regions.

#### 2.7.2 Heatmap clustering

The reduced heatmap was generated using hierarchical clustering in seaborn.clustermap. The final heatmap used Ward linkage, Euclidean distance, and k = 4 clusters for both samples and signatures. Before clustering, all selected signatures were standardised using StandardScaler, so the heatmap reflected z-scored values rather than raw contributions. The output also included row and column cluster assignments, cluster boundaries, and a cross-tabulation of sample clusters versus response group.

### 2.8 Standardised median difference

For each retained signature, a standardised median difference (standardised Δ) was computed between the adverse-outcome and favourable outcome groups:


$$\text{Δ}=\frac{\text{median}\left(\text{adverse}\right)-\text{median}\left(\text{favourable}\right)}{\text{global SD}}$$

where global SD is the standard deviation of the signature contribution across the full cohort. Under this convention, positive values denote enrichment in the adverse-outcome group, and negative values denote enrichment in the favourable-outcome group. Between-group comparisons were performed with a two-sided Mann-Whitney U test. A 95% confidence interval for each standardised Δ was obtained by bootstrap resampling with 2,000 iterations, taking the 2.5^th^ and 97.5^th^ percentiles of the resulting distribution. To account for simultaneous testing across signatures, nominal p-values were adjusted with the Benjamini–Hochberg method, and the resulting false discovery rate (FDR) is reported alongside each p-value. For display, signatures were ranked by a composite score that combines effect size and group separation:


$$\text{score}=-{\text{log}}_{10}\left(p\right)\times |\text{Δ}|$$

**Figure 1:**
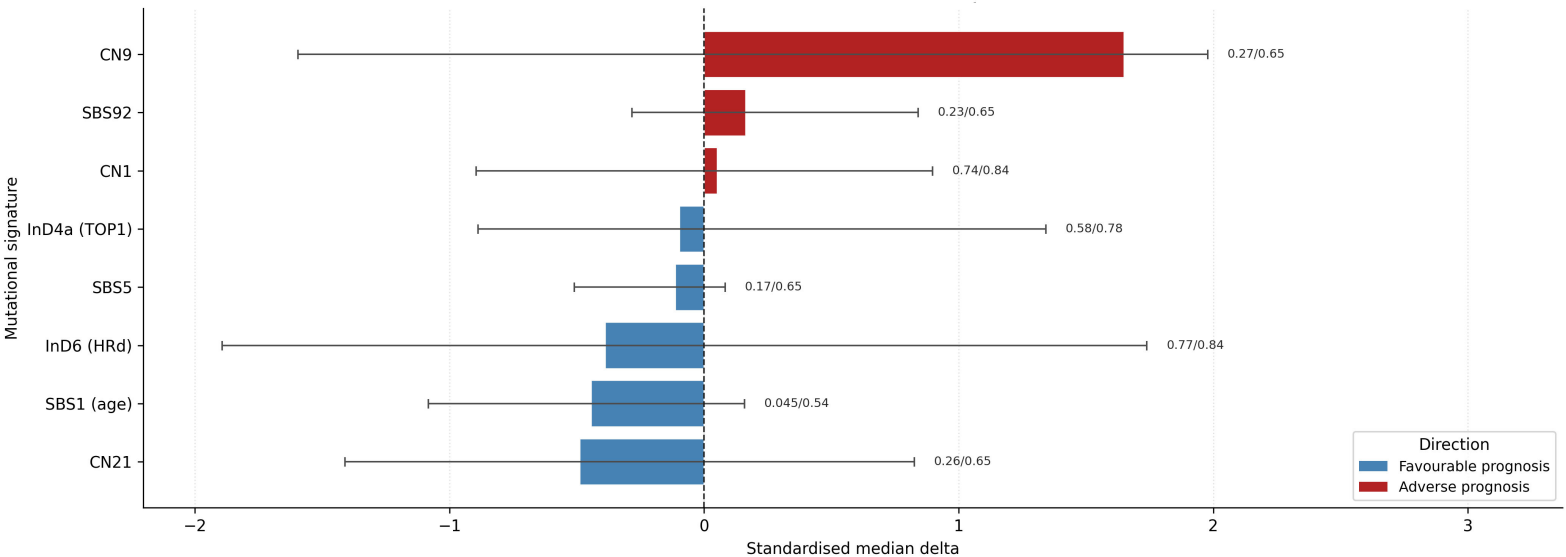
Comparison of clinical prognosis against genomic signatures using the standardised median difference (standardised Δ). The horizontal bar plot displays the standardised Δ for eight selected molecular signatures, calculated as the standardised median difference in signature contribution between favourable-outcome and adverse-outcome patients. Positive values (red, right) indicate higher contribution in the adverse-outcome group; negative values (blue, left) indicate higher contribution in the favourable-outcome group. The vertical dashed line marks the null reference (Δ = 0). Error bars represent bootstrap 95% confidence intervals. The values to the right of each bar are the two-sided Mann–Whitney U p-value and the Benjamini–Hochberg adjusted false discovery rate (p/FDR). This analysis is exploratory.

The eight signatures with the highest score are presented in the ranked plot of standardised Δ (**Figure 1**).

### 2.9 Statistical analyses

All statistical analyses were exploratory. Clinical and genomic variables were described through absolute and relative frequencies for categorical variables, and median and range for continuous variables. Categorical variables were compared with the χ^2^ or Fisher’s exact test, as appropriate. Survival was analysed by the Kaplan–Meier method and compared with the log-rank test. Hazard ratios were obtained by Cox regression when the number of events and the sample size allowed. Missing data were handled by variable-by-variable exclusion without imputation, and the number of evaluable cases was reported for each analysis. Clinical statistical and survival analyses were performed in SPSS v28. Median contributions of SBS, indel and CN signatures across the four clinicogenomic groups were compared descriptively. The signature-level comparison between favourable-outcome and adverse-outcome patients, including the standardised median difference, its confidence interval and the multiple-testing correction, is described in the preceding section.

### 2.10 Ethical considerations

The study was conducted in accordance with the Declaration of Helsinki and was approved by the Research Ethics Committee of the University Hospital of Salamanca, protocol code PI 2022 10 1155, approval date 29 November 2022. All patients provided written informed consent for sample collection, molecular analysis and the use of clinical and genomic data for research purposes.

### 2.11 Data availability

The datasets generated and analysed during the current study are available in the Sequence Read Archive (SRA) repository [submission: SUB15983278]. Accession to cite for these SRA data: PRJNA1424539 (https://www.ncbi.nlm.nih.gov/sra/PRJNA1424539).

## 3 Results

### 3.1 Clinical and genomic characteristics of the cohort

A total of 37 patients with advanced NSCLC were included (**Supplementary Table 1**). All patients had baseline ctDNA collected before the start of first-line treatment and analysed by WES. The cohort comprised 25 patients with adenocarcinoma (67.6%), 10 with squamous-cell carcinoma (27.0%), and 2 with undifferentiated carcinoma (5.4%). Seventeen patients had PD-L1 expression ≥50%, and 20 had PD-L1 expression<50%. Median PFS in the overall cohort (from now on, the cohort) was 9 months, and median OS was 20 months. The distribution of patients across the four clinicogenomic groups is presented in **Table 1**.

**Table 1:** Clinicogenomic groups according to PD-L1 expression and PFS.

Group	Clinical definition	n	Median PFS (months)	Median OS (months)
1	PD-L1 ≥50% and adverse outcome	9	4	10
2	PD-L1<50% and adverse outcome	11	8	12
3	PD-L1<50% and favourable outcome	9	16	24
4	PD-L1 ≥50% and favourable outcome	8	22	22

PD-L1, programmed death-ligand 1; PFS, progression-free survival; OS, overall survival.

Median TMB-SNV was 7.20 mutations/Mb in patients with adverse outcome and 6.79 mutations/Mb in patients with favourable outcome. Ten patients had TMB-SNV values above 10 mutations/Mb; 5 belonged to the adverse-outcome group and 5 to the favourable-outcome group. According to PD-L1 status, median TMB-SNV was 7.18 mutations/Mb in patients with PD-L1<50% and 6.79 mutations/Mb in patients with PD-L1 ≥50%. bTMB values tended to be higher in patients with adverse outcome, in some cases exceeding 10 mutations per megabase, whereas patients with favourable outcome generally had lower or moderate values. TMB-SNV values were similar across outcome and PD-L1 subgroups, with no meaningful difference observed between groups.

### 3.2 Integrated genomic signature analysis

**Figure 2:**
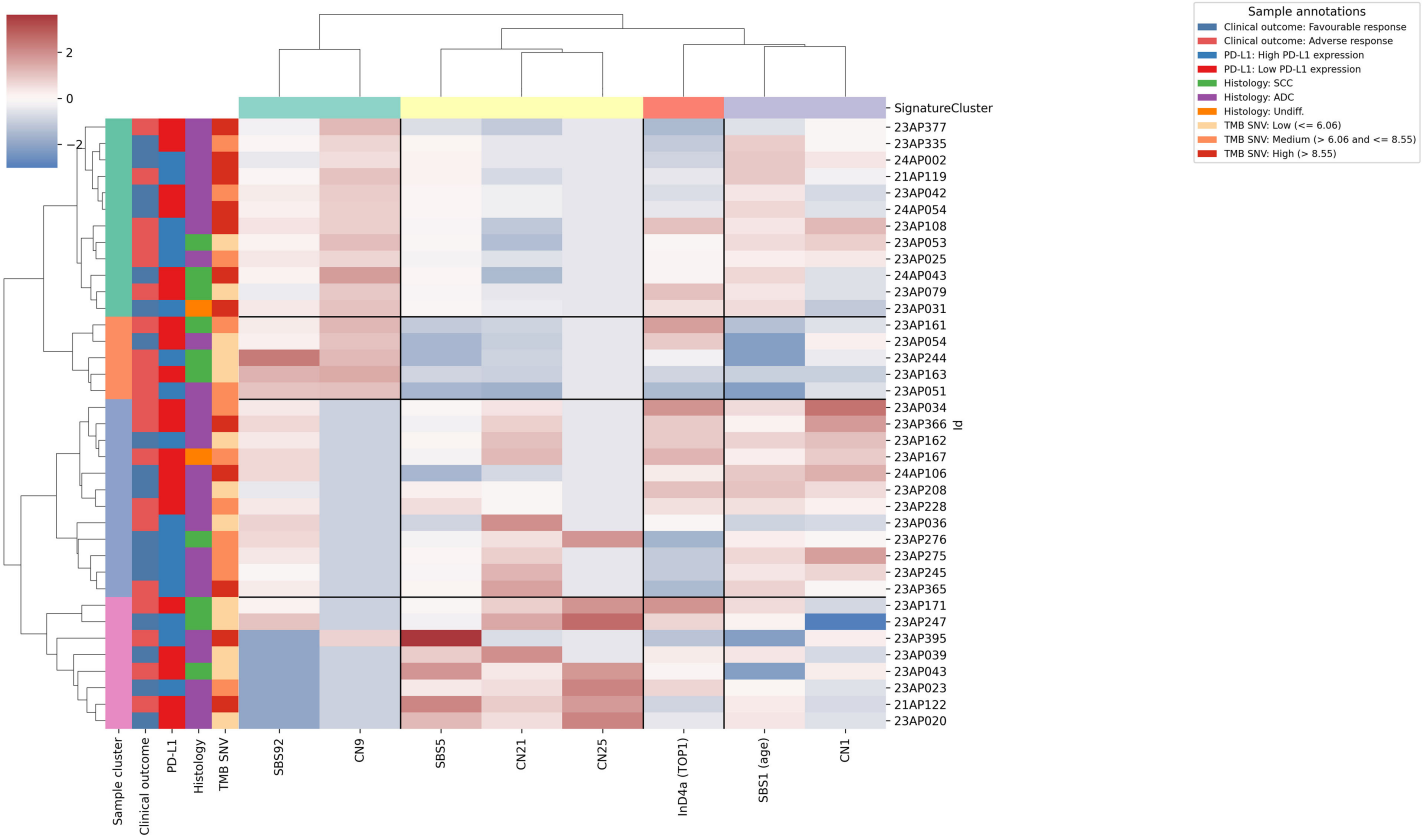
Integrated heatmap of selected ctDNA-derived genomic signatures across the cohort (n = 37). Rows correspond to samples and columns to single-base substitution, indel and copy-number signatures. Hierarchical clustering was applied on both axes. The colour scale represents signature contributions after row standardisation (z-scores), from low (blue) to high (red). The top colour bar (SignatureCluster) indicates the signature modules defined by clustering. The five lateral bars annotate, for each sample: sample cluster; clinical outcome (favourable-outcome, blue; adverse-outcome, red); PD-L1 expression (high, blue; low, red); histology (squamous-cell carcinoma [SCC], green; adenocarcinoma [ADC], purple; undifferentiated [Undiff.], orange); and tumour mutational burden by single-nucleotide variants (TMB-SNV: low ≤6.06; medium >6.06 and ≤8.55; high >8.55 mutations/Mb). For biological context, the displayed signatures correspond to the following processes: SBS1 and SBS5, clock-like signatures linked to ageing; SBS92, tobacco-like in this dataset; InD4a (TOP1), topoisomerase I-associated; CN1, near-diploid genome; CN9, loss of heterozygosity and chromosomal instability; CN21, aetiology not yet established; CN25, a minor signature shown without individual annotation. This analysis is exploratory and the clustering is descriptive.

The unsupervised hierarchical clustering using the contributions of SBS, InD, and CN signatures revealed four major sample clusters (C1–C4) and four signature modules characterised by distinct co-occurrence patterns (**Figure 2**).

Cluster C1 comprised the majority of the samples and displayed relatively homogeneous mutational profiles, characterised by moderate contributions of SBS92 and CN9, and low contributions of signatures associated with HRD, including InD6, CN21, and CN25. Cluster C2 consisted of a single sample, which segregated from the remainder of the cohort due to an exceptionally high contribution of CN20, resulting in its early separation in the hierarchical dendrogram. Cluster C3 exhibited a distinct profile, enriched for SBS5, InD6, CN21, and CN25. Finally, cluster C4 displayed a pattern characterised by increased contributions of CN1, CN23, and InD4a. Notably, several samples showed particularly high contributions of CN23, contributing to greater intracluster heterogeneity. Clustering of the signatures identified four major modules: the first included SBS5, InD6, CN21, and CN25, the second comprised CN20, SBS92, and CN9, the third grouped SBS1 and InD10, and the fourth contained InD4a, CN1, and CN23.Prognostic analysis based on genomic signatures (standardised Δ).

### 3.3 Prognostic analysis based on genomic signatures (standardised Δ)

The standardised median difference (standardised Δ) was calculated for each identified signature to evaluate the association between the different molecular signatures and patient prognosis (**Figure 1**). The CN9 signature showed the largest standardised Δ in the adverse direction (Δ = 1.625), whilst CN21, SBS1, and InD6 signatures exhibited the most substantial favourable impact, clustering with median Δ values between -0.4 and -0.5.

### 3.4 SBS mutational signatures

The SBS signatures evaluated were SBS1, SBS92, SBS5, SBS3, SBS2, SBS11, SBS17, SBS30 and SBS95. The dominant signatures in the cohort were SBS1, SBS5 and SBS92. SBS5 was detected in 33/37 patients, with a median contribution value of 22. SBS1 was detected in 32/37 patients, with a median exposure value of 31. SBS92 was detected in 31/37 patients, with a median contribution value of 38. The combined exposure of SBS1 + SBS5 yielded a median value of 55 in the cohort.

Across samples, the mutational profile was predominantly characterised by the contribution of clock-like signatures SBS1 and SBS5, consistent with endogenous mutational processes associated with ageing. In contrast, the HRD-associated signature SBS3 was detected only in a limited number of cases and generally at low contribution levels. Similarly, APOBEC-related activity (SBS2) was largely absent, with only sporadic detection in individual samples. Other signatures, including SBS11, SBS17, SBS30, and SBS95, showed minimal contributions. When analysed according to clinical outcome, median SBS exposure values were similar between groups. Across the clinicogenomic groups, the distribution of the main SBS signatures was also comparable (**Table 2**).

**Table 2:** Summary of SBS mutational signatures.

SBS signature	Biological interpretation	Detected, n/N	Overall median exposure (range)	Adverse outcome median	Favourable outcome median	G1 median	G2 median	G3 median	G4 median
SBS1	Age/clock-like	32/37	31 (0 –38)	29	33	29	29	34	32
SBS92	Tobacco-related/tobacco-like in this dataset	31/37	38 (0 –73)	38.5	37	40	38	36	39
SBS5	Clock-like basal process	33/37	22 (0 –76)	21	23	21	21	23	22.5
SBS3	HRD-associated	6/37	0 (0 –39)	0	0	0	0	0	0
SBS2	APOBEC-associated	3/37	0 (0 –15)	0	0	0	0	0	0
SBS11	Alkylating-agent associated	1/37	0 (0 –20)	0	0	0	0	0	0
SBS17	Unknown/oxidative or therapy-related context	3/37	0 (0 –20)	0	0	0	0	0	0
SBS30	Base-excision repair-related context	1/37	0 (0 –32)	0	0	0	0	0	0
SBS95	Deamination-related context	1/37	0 (0 –31)	0	0	0	0	0	0

Values are signature exposure values generated by the bioinformatic pipeline. Medians refer to exposure values, not to absolute mutation counts or mutations per megabase. SBS, single-base substitution; HRD, homologous recombination deficiency; APOBEC, apolipoprotein B mRNA editing catalytic polypeptide-like; G1, clinicogenomic group 1; G2, clinicogenomic group 2; G3, clinicogenomic group 3; G4, clinicogenomic group 4.

### 3.5 InD mutational signatures

The InD signatures evaluated were InD4a, InD10, InD6 and InD3a. InD4a, associated with TOP1 activity, showed variability across samples, with elevated levels observed in patients with adverse outcome. InD10 exhibited a wide range in both outcome groups, with no clear association with clinical outcome. InD6, associated with HRD, was absent in most cases and showed isolated occurrences in both groups, suggesting a limited contribution in this cohort. InD3a, associated with tobacco-related mutagenesis, was largely absent, with a few isolated positive values. No InD signature showed a consistent or meaningful difference between adverse- and favourable-outcome patients.

### 3.6 CN signatures

**Figure 3:**
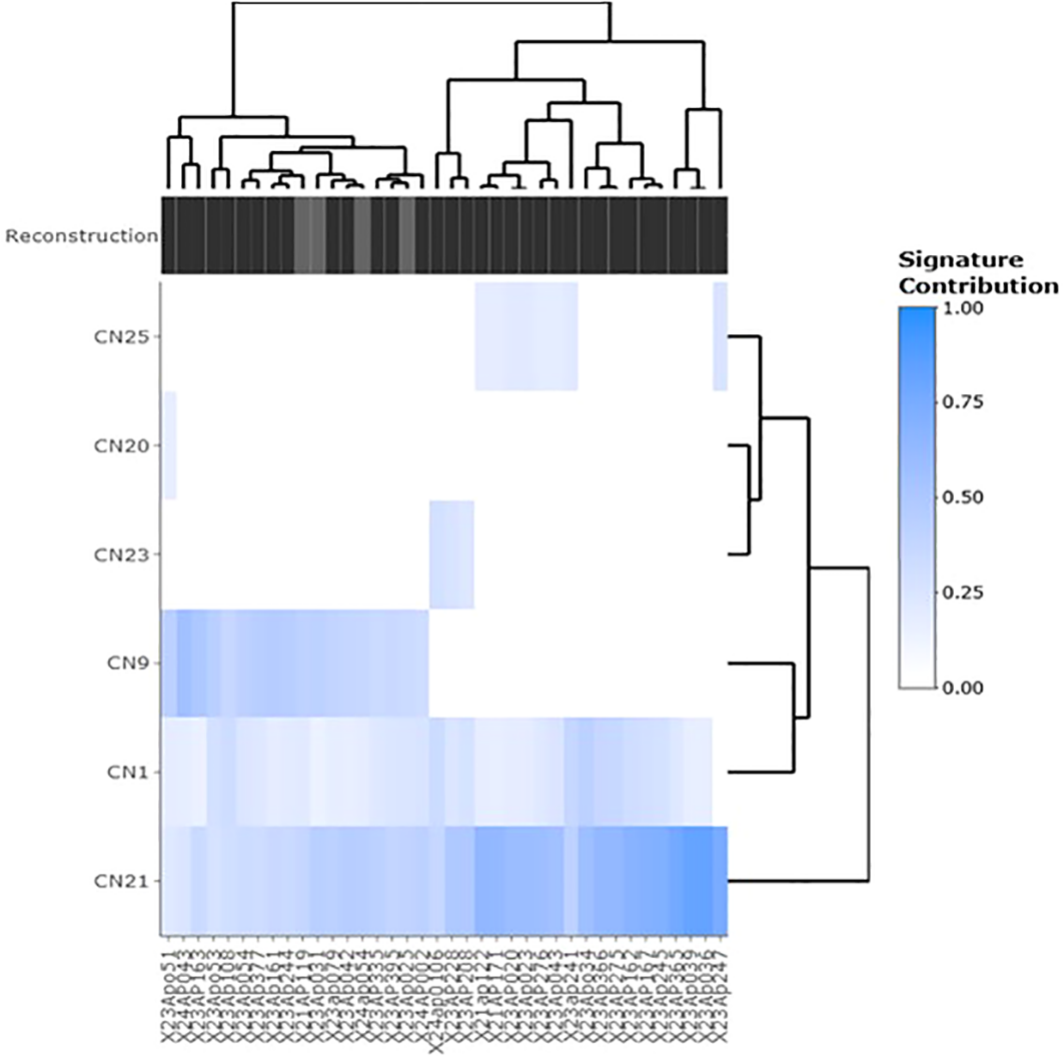
Hierarchical clustering of COSMIC copy-number (CN) signatures. Heatmap displaying the activity of CN signatures across the analysed samples. Unsupervised hierarchical clustering identified distinct groups of tumours with similar CN signature profiles. Increased contributions of signatures such as CN9 and CN21 were observed in subsets of samples, suggesting the presence of diverse chromosomal instability processes within the cohort. Colour scale indicates relative signature activity. This analysis is exploratory.

The distribution of CN signature activities across the cohort was visualised using a heatmap with hierarchical clustering of both samples and signatures (**Figure 3**). The CN signatures evaluated were CN1, CN9, CN20, CN21, CN23, and CN25. In the cohort, CN21 was detected in 37/37 patients, representing the highest contribution within individual tumours and the highest median value: 45.1. CN1 was detected in 36/37 patients, with a median exposure value of 22.8. CN9 was detected in 18/37 patients, with a median exposure value of 31.3. CN20, CN23 and CN25 were detected in 1/37, 3/37 and 7/37 patients, respectively.

When analysed according to clinical outcome, CN9 showed the largest difference between groups: it was detected in 11/20 adverse-outcome patients and 7/17 favourable-outcome patients with a median exposure of 35 in the former and 0 in the latter. CN21 was present in all the cohort, with a median exposure of 41.3 in patients with adverse outcome and 49.5 in those with favourable outcome. CN1 showed similar median exposure values between groups. Among adverse-outcome patients, CN9 was dominant in 8/20 cases, compared with 2/17 cases amongst favourable-outcome patients. The CN signature profile was mainly represented by CN21 and CN1, whilst CN9 identified a more restricted subgroup of patients. CN9 showed higher exposure in the adverse-outcome group, whereas CN21 showed higher median exposure in the favourable-outcome group.

Across the four clinicogenomic groups, median CN9 exposure was highest in Group 1, with a value of 36.7. Median CN9 contribution was 0 in Group 2, 34.6 in Group 3 and 0 in Group 4. Median CN21 exposure was 37.8 in Group 1, 56.1 in Group 2, 44.8 in Group 3 and 61.7 in Group 4. CN21 therefore showed the highest median exposure in Group 4.

**Figure 4:**
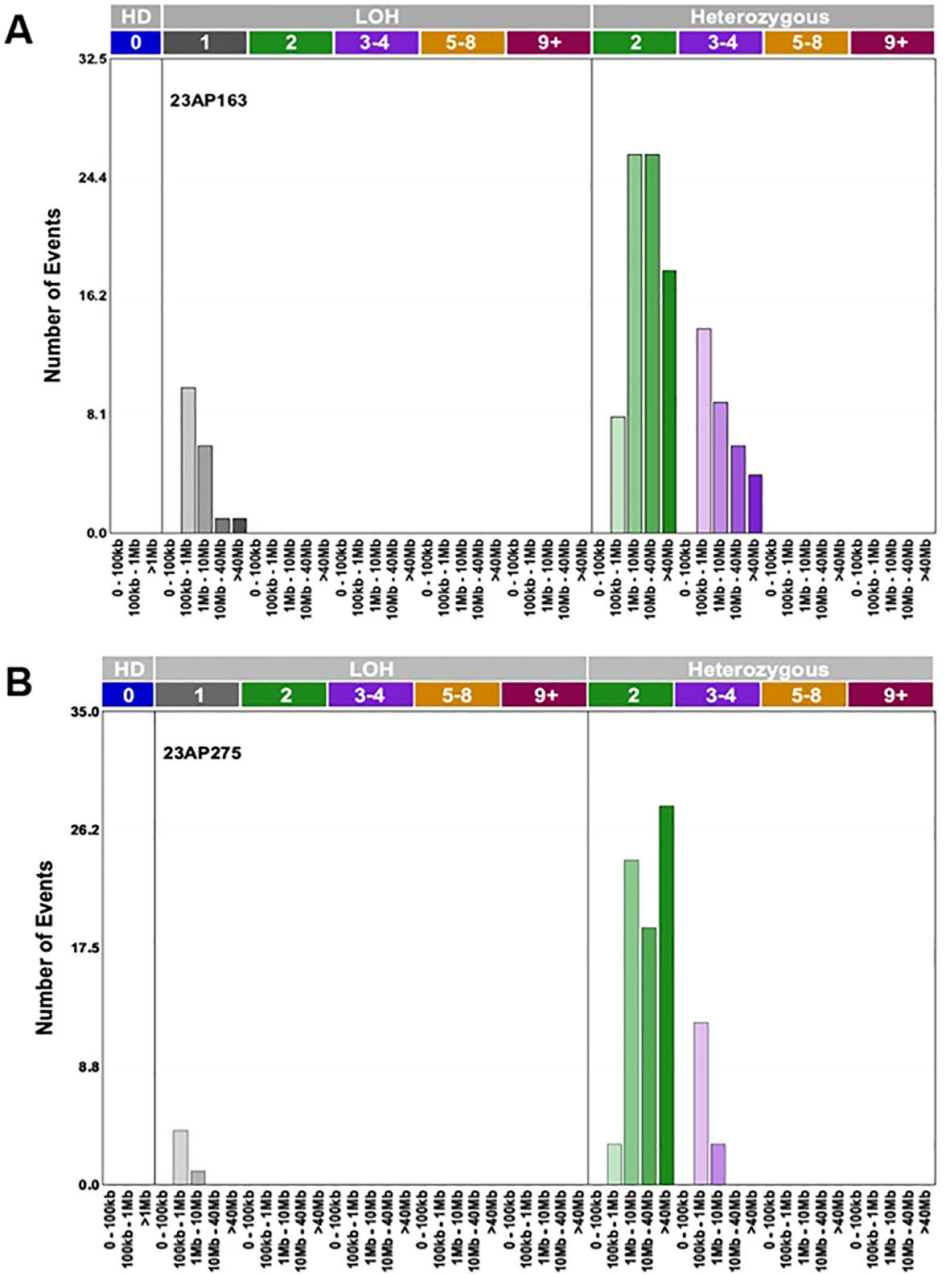
Copy-number (CN) feature profiles of a favourable-outcome **(A)** and an adverse-outcome **(B)** case used for COSMIC CN signature assignment. Each histogram displays the distribution of genomic segments according to segment size, heterozygosity status, and total CN state, with events categorised as loss of heterozygosity (LOH) or heterozygous and stratified by CN class (2, 3–4, 5–8, and ≥9 copies). **(A)** Sample 23AP275 (patient 27 of the dataset, **Supplementary Table 1**) corresponds to a lung adenocarcinoma with high PD-L1 expression, showing a profile dominated by heterozygous segments of 2–4 copies with infrequent LOH events; CN exposures were CN21 (63.8%) and CN1 (36.2%), without CN9. This patient achieved a partial response, with a progression-free (PFS) of 22 months. **(B)** Sample 23AP163 (patient 19) corresponds to a squamous-cell carcinoma with low PD-L1 expression, exhibiting a higher burden of LOH events; CN exposures were CN9 (50.4%), a signature associated with focal LOH and chromosomal instability, and CN21 (34.1%), of currently unknown aetiology. This patient had no response to treatment; PFS of 3 months. These two profiles are presented as representative examples of contrasting CN signature compositions at the individual-sample level, and do not constitute evidence of an independent association between CN signatures and clinical outcome.

Representative CN feature profiles of a favourable-outcome and an adverse-outcome patient are shown in **Figure 4**.

## 4 Discussion

In this exploratory study, ctDNA-derived CN signatures showed the most apparent separation between outcome groups amongst the signature classes examined, although none of these differences reached statistical significance after correction for multiple testing. The mutational landscape was heterogeneous and dominated by endogenous processes, with limited contribution from specific DNA repair deficiencies or exogenous mutagens. The most consistent observation was the opposite direction of two CN signatures. CN9 tended towards enrichment in patients with adverse outcome, whereas CN21 reached higher exposure in patients with better disease control. This contrast was most apparent amongst patients with PD-L1 ≥50%, where two distinct subsets emerged: Group 1, with early progression and higher CN9 exposure, and Group 4, with prolonged PFS, absent or low CN9, and higher CN21 exposure. These two signatures plausibly reflect different mechanisms of genome maintenance: CN21-enriched tumours may relate to whole-genome doubling and ongoing chromosomal instability, whilst CN9-dominated profiles may arise from focal rearrangement processes such as replication stress or breakage–fusion–bridge cycles (8, 23).

These results are consistent with the literature supporting that PD-L1 and TMB are incomplete predictive biomarkers for ICI outcomes in advanced NSCLC. Although TMB has been proposed as a surrogate of neoantigen load, its predictive value remains uncertain. The B-F1RST trial suggested that bTMB may enrich for benefit from atezolizumab (24), whereas the BFAST cohort C trial did not confirm an independent predictive role for bTMB (25). In our cohort, TMB-SNV was very similar between patients with adverse and favourable outcomes, and patients with TMB-SNV >10 mutations/Mb were evenly distributed between both groups. This finding supports the view that mutation burden alone is insufficient to account for differential benefit from ICI.

The SBS profile of our cohort was mainly represented by SBS1 and SBS5. The predominance of these clock-like signatures is consistent with prior mutational signature NSCLC studies, where they have been found to reflect endogenous ageing-related mutational processes and have been consistently observed across multiple cancer types (20). The widespread presence of these signatures in our cohort likely reflects the contribution of aging-related mutagenesis to tumour evolution. In contrast, signatures associated with APOBEC activity or homologous recombination deficiency, such as SBS2 and SBS3, were uncommon in our series. APOBEC-related activity was restricted to a subset of tumours, suggesting that cytidine deaminase-mediated mutagenesis contributes to tumour evolution in selected patients rather than representing a universal feature of NSCLC. Previous studies have linked APOBEC activity with increased neoantigen generation and modulation of antitumour immune responses (10, 26). Although APOBEC-related activity has been associated with tumour evolution (27, 28) and, in some studies, with ICI response (10), it did not appear to be a dominant process in our cohort. SBS signatures therefore provided biological context but did not clearly explain the clinical differences between favourable and adverse outcomes.

Likewise, InD signatures were not the main discriminatory component. InD4a and InD10 predominated, whilst InD6, associated with HRD, was detected only in a minority of patients. This observation is consistent with previous genomic studies demonstrating that classical HRD phenotypes occur less frequently in NSCLC than in breast or ovarian cancers (29). Nevertheless, emerging evidence across solid tumours suggests that even low levels of DNA repair deficiency may influence tumour immunogenicity and response to ICI (30, 31).

The most distinctive findings of this study came from CN signatures. Earlier work by Macintyre and colleagues (32), and later by Steele and colleagues (8), extended the signature framework to CN alterations and showed that structural genomic patterns can reflect different mechanisms of chromosomal instability and tumour evolution. Our results aligned with this concept. CN signatures captured clinically relevant heterogeneity that was not evident from TMB or SBS and InD signatures alone. CN21 was the dominant CN signature in most patients, whilst CN9 identified a smaller subgroup with poorer outcome.

The clinical relevance of CN9 lies in its potential ability to identify patients with primary resistance despite high PD-L1 expression. In Group 1, patients had PD-L1 ≥50% but a median PFS of 4 months together with the highest CN9 exposure, suggesting that CN9 may reflect a structural genomic state linked to early immune escape. CN21 showed the opposite pattern, reaching its highest exposure in Group 4, where patients had PD-L1 ≥50%, favourable outcome, absent or low CN9, and the longest median PFS. Although the biological significance of these signatures remains to be fully elucidated, accumulating evidence indicates that large-scale chromosomal alterations and CN architecture can modulate antitumour immunity and influence responsiveness to ICI (14, 33).

From a clinical perspective, these findings support the development of composite biomarker models integrating PD-L1, TMB and ctDNA-derived CN signatures (13). The main value of this study is not to propose CN9 or CN21 as validated standalone biomarkers, but to show that ctDNA-WES can identify structural genomic phenotypes that help explain early resistance or unexpected benefit in advanced NSCLC treated with ICI.

Our study had several limitations. The sample size was small, the design was exploratory and single-centre, and no external validation was performed; consequently, no signature retained significance after correction for multiple testing. Signatures were assessed only at baseline, without longitudinal ctDNA sampling at progression. Because all signatures were derived from plasma cfDNA, several technical factors constrained the CN results. Reduced tumour fraction lowered sensitivity for subclonal and focal CN events, and exposures inferred from low-input plasma were subject to greater sampling variability than tissue-derived estimates. No replicate sequencing or orthogonal validation was performed, so the technical reproducibility of the ctDNA-derived CN signatures was not formally assessed. Matched tumour tissue was not analysed either, and it remains undetermined whether the same conclusions would hold in tissue, given ctDNA-specific factors such as fragmentation bias, plasma background, and clonal heterogeneity. Within these constraints, our results suggest that ctDNA-derived CN signatures may capture clinically relevant resistance patterns beyond PD-L1 and TMB, with CN9 as a candidate resistance-associated signature and CN21 as a possible marker of more favourable disease control. These hypotheses require testing in larger prospective and multicentre cohorts.

## 5 Conclusions

In this exploratory cohort of patients with advanced NSCLC treated with first-line ICI, PD-L1 expression and TMB did not fully account for clinical outcome. ctDNA-derived CN signatures provided additional information: CN9 was enriched in patients with adverse outcome, notably amongst those with PD-L1 ≥50% and early progression, whereas CN21 was more prominent in patients with prolonged disease control. These findings suggest that structural genomic signatures obtained from ctDNA-WES may represent an axis of genomic instability complementary to PD-L1 and TMB, and could help refine biomarker models in this clinical scenario. Validation in larger prospective and multicentre cohorts, ideally incorporating longitudinal ctDNA sampling, is required before clinical implementation.

## Data Availability

The datasets generated and analysed during the current study are available in the Sequence Read Archive (SRA) repository (submission: SUB15983278). Accession to cite for these SRA data: PRJNA1424539 (https://www.ncbi.nlm.nih.gov/sra/PRJNA1424539).

## Supplementary material

Supplementary Table 1 Patient-level clinical, demographic, and genomic dataset for the full cohort (n = 37). Columns include clinical and demographic variables, programmed death-ligand 1 (PD-L1) status, first-line treatment, objective response, progression-free survival (PFS), overall survival (OS), clinicogenomic cluster, tumour fraction, single-nucleotide-variant-derived TMB (TMB-SNV), and exposure values for all evaluated single-base substitutions (SBS), indels (InD), and copy-number (CN) signatures. Signature values are exposures generated by the bioinformatic pipeline, not absolute mutation counts.
